# Psychische Gesundheit und psychiatrisch-psychotherapeutische Versorgung als Elemente von „pandemic and crisis preparedness“

**DOI:** 10.1007/s00115-025-01822-w

**Published:** 2025-04-01

**Authors:** Hauke Felix Wiegand, Kristina Adorjan, Jutta Stoffers-Winterling, Simone Scheithauer, Jochen Schmitt, Oliver Tüscher, Peter Falkai, Klaus Lieb

**Affiliations:** 1 Universitätsklinik und Poliklinik für Psychiatrie, Psychotherapie und Psychosomatik, Universitätsmedizin Halle, Martin-Luther-Universität Halle-Wittenberg, Julius-Kühn-Straße 7, 06112 Halle (Saale), Deutschland; 2 Klinik für Psychiatrie und Psychotherapie, Universitätsmedizin Mainz, Johannes Gutenberg-Universität Mainz, Mainz, Deutschland; 3 Klinik für Psychiatrie und Psychotherapie, Universität Bern, Bern, Schweiz; 4 Klinik für Psychiatrie und Psychotherapie, LMU Klinikum, Ludwig-Maximilians-Universität München, München, Deutschland; 5 Leibniz Institut für Resilienzforschung (LIR), Mainz, Deutschland; 6 Institut für Hygiene und Infektiologie, Universitätsmedizin Göttingen, Göttingen, Deutschland; 7 Zentrum für Evidenzbasierte Gesundheitsversorgung, Universitätsklinikum und Medizinische Fakultät Carl Gustav Carus, Technische Universität Dresden, Dresden, Deutschland

**Keywords:** COVID-19, Versorgungssurveillance, Risikogruppen, Gesundheitspersonal, Finanzierungsmodelle, COVID-19, Health surveillance, Risk groups, Healthcare personnel, Financing models

## Abstract

**Hintergrund:**

Die COVID-19(„coronavirus disease 2019“)-Pandemie war herausfordernd bezüglich der psychischen Gesundheit der Bevölkerung und der psychiatrisch-psychotherapeutischen Versorgung.

**Ziele der Arbeit:**

Erkenntnisse aus der Pandemie für eine verbesserte Vorbereitung auf zukünftige Krisen („pandemic and crisis preparedness“) gewinnen.

**Material und Methoden:**

Aus den Ergebnissen von Reviews zu psychischer Gesundheit und psychiatrisch-psychotherapeutischer Versorgung in der Pandemie werden Empfehlungen abgeleitet.

**Ergebnisse:**

Große Teile der Bevölkerung erwiesen sich in ihrer psychischen Gesundheit als resilient. Risikopopulationen zeigten erhöhte Belastungen, insbesondere Kinder, Jugendliche, Frauen, Menschen mit niedrigem soziökonomischem Status, Menschen in Heimen sowie Gesundheitspersonal. Besonders betroffen waren auch Menschen mit Long‑/Post-COVID. Gleichzeitig kam es zu signifikanten Einschränkungen der psychiatrisch-psychotherapeutischen Versorgung, insbesondere im stationären Bereich. Eine unzureichende aktuelle Datenlage zu psychischer Gesundheit und fehlende Versorgungssurveillance – trotz existierender Datengrundlage – erschwerte die rechtzeitige Detektion von Belastungen und Versorgungseinschränkungen sowie adäquate Reaktionen zur Krisenbewältigung. Die starre Sektorisierung des Versorgungssystems verhinderte vielfach eine alternative ambulantisierte Versorgung.

**Diskussion:**

Die Nationale Mental Health Surveillance sollte weitergeführt und -entwickelt werden. Es sollten Strukturen zur zeitsensitiven Zusammenführung von Daten sowie multiprofessionellen Generierung von Empfehlungen vorgehalten werden, um Versorgungsengpässe und Belastungen von Risikogruppen detektieren und darauf reagieren zu können. Flexible, sektorübergreifende Versorgung sollte deutschlandweit ermöglicht werden.

## Abstract (en)

**Background:**

The COVID-19 pandemic was challenging in terms of the mental health of the population and the provision of mental health services.

**Aims of the study:**

To gain insights from the pandemic for an improved pandemic and crisis preparedness for the future.

**Material and methods:**

Recommendations are derived from the results of reviews on mental health and psychiatric psychotherapeutic care during the pandemic.

**Results:**

Large sections of the population proved to be resilient in terms of mental health. Populations at risk showed increased stress, particularly children, adolescents, women, people with low socioeconomic status, people in nursing homes and healthcare workers. People with long/post-COVID were also particularly affected. At the same time there were significant restrictions on the provision of psychiatric psychotherapeutic care, particularly in the inpatient sector. Insufficient current data on the mental health of the population and a lack of surveillance of service utilization, despite an existing data basis, made it difficult for timely detection of stress and care restrictions and an adequate response for crisis management. The rigid sectorization of the mental healthcare system often prevented alternative outpatient care.

**Discussion:**

The national mental health surveillance should be continued and further developed. Structures should be provided for the time-sensitive consolidation of data and the multiprofessional generation of recommendations in order to be able to detect and respond to challenges due to bottlenecks in the provision of care and stress in risk groups. Flexible, cross-sectoral mental healthcare should be made possible throughout Germany.

## Hintergrund

Die COVID-19(„coronavirus disease 2019“)-Pandemie war eine für die Gesundheit der Bevölkerung und das Gesundheitssystem herausfordernde Zeit nicht nur bezüglich der hohen Inzidenzen von SARS-CoV-2(„severe acute respiratory syndrome coronavirus type 2“)-Infektionen, sondern auch bezüglich der psychischen Gesundheit der Bevölkerung und im Hinblick auf die Aufrechterhaltung einer adäquaten Gesundheitsversorgung. Wie im narrativen Review von Stoffers-Winterling et al. beschrieben, zeigte sich in der ersten, besonders gut untersuchten Pandemiephase zwar ein großer Teil der Bevölkerung resilient gegenüber der Krisenlage, ab dem Jahr 2022 gab es dann Hinweise auf einen Anstieg der psychischen Belastung in der Bevölkerung. Insbesondere für Risikogruppen zeigte sich jedoch bereits ab dem Winter 2020 ein Zuwachs der Belastung [27]. Bezüglich der psychiatrisch-psychotherapeutischen Versorgung konnten der systematische Review mit Metaanalyse von Erdekian et al. zusammenfassen, zu welchen signifikanten Einschränkungen insbesondere der stationären psychiatrisch-psychotherapeutischen Versorgung es in der COVID-19-Pandemie kam [7].

Diese Arbeit diskutiert im Folgenden, wie die Ergebnisse der Evidenzsynthesen von Stoffers-Winterling et al. und Erdekian et al. zu bewerten sind und welche Schlussfolgerungen sich für eine verbesserte Vorbereitung auf zukünftige Pandemien („pandemic and crisis preparedness“) im Hinblick auf die psychische Gesundheit der Bevölkerung und auf eine resilientere Gesundheitsversorgung in den Psych-Fächern (Psychiatrie, Psychotherapie, Psychosomatik, Kinder- und Jugendpsychiatrie und Psychotherapie) ziehen lassen.

## Psychische Gesundheit der Bevölkerung

Im Hinblick auf die psychische Gesundheit der Bevölkerung und auf die von spezifischen Risikogruppen wie z. B. des Gesundheitspersonals war insbesondere herausfordernd, dass initial vielfach ein Baseline-Datensatz fehlte, um Veränderungen im Rahmen der Pandemie abzubilden. Teils konnten Ergebnisse von Kohortenstudien wie beispielsweise der NAKO-Gesundheitsstudie, der GEDA-Studie des Robert-Koch-Instituts (RKI), der LORA-Studie oder für Kinder und Jugendliche der COPSY-Studie, welche an die leider nicht fortgeführte BELLA/KiGGS-Studie anschließt, verwendet werden. Daher war insbesondere die Etablierung der – leider weiter langfristig in der Fortführung gefährdeten – Nationalen Mental Health Surveillance des RKIs ein wichtiger Schritt, um langfristige, repräsentative Daten zumindest für die Allgemeinbevölkerung vorzuhalten [15, 30].

## Psychische Gesundheit von Risikogruppen

Bestimmte Risikopopulationen waren in der Pandemie besonders durch einen Zuwachs der psychischen Belastung betroffen. Zu diesen gehörten insbesondere Kinder- und Jugendliche, Frauen und sozioökonomisch benachteiligte Personen [27]. Maßnahmen zum Infektionsschutz wie Schließungen von Schulen, Kindergärten und Sportvereinen, hochgradige Kontakteinschränkungen sowie die Einstellung tagesstrukturierender Gruppenangebote in Heim- und Wohneinrichtungen waren angesichts der Infektionsausbreitung in vulnerablen Populationen in der hoch dynamischen und mit großen Unsicherheiten behafteten Situation der Pandemie gut nachvollziehbar. Andererseits lässt sich aus diesen Entwicklungen ableiten, dass die Effekte dieser Maßnahmen auf die psychische Gesundheit, z. B. die Vereinsamung durch fehlende soziale Kontakte oder Belastungen durch die Kompensation wegfallender Kinderbetreuungsangebote, rascher erfasst werden müssen und die wissenschaftliche Diskussion sowie die Politikberatung entsprechend multiperspektivisch aufzustellen sind, wie unter anderem auch vom Sachverständigenrat Gesundheit und Pflege empfohlen [20]. Auch das Leibniz-Lab „Pandemic Preparedness“, ein Netzwerk aus 41 Leibniz-Instituten, arbeitet an Konzepten zur Vorbeugung und Bewältigung psychischer Belastungen während Pandemien in besonders belasteten Personengruppen wie Schülern [13].

## Psychische Gesundheit von Gesundheitspersonal

Wie ebenfalls von Stoffers-Winterling et al. beschrieben, zeigte sich in Studien und Metaanalysen zur psychischen Belastung des Gesundheitspersonals in der initialen Phase der Pandemie kein allgemeiner Anstieg der Belastung in dieser Gruppe [27]. Ergebnisse z. B. der LORA-Studie [1] sowie aus einem systematischen Review [21] wiesen jedoch darauf hin, dass Risikogruppen differenzierter betrachtet werden müssen. Belastungen durch geänderte Arbeitsbedingungen, Infektionsschutzmaßnahmen, psychische Gesundheit und Absentismus bzw. „intention to leave“ und „intention to change“, also die Absicht von Gesundheitspersonal, einer anderweitigen Beschäftigung nachzugehen, zeigten relevante Assoziationen [19, 25]. Dies ist insofern von Bedeutung, als der Mangel an qualifiziertem Personal eine der zentralen Herausforderungen des Managements der COVID-19-Pandemie war [4]. Hervorzuheben war außerdem der als Reaktion auf moralische Konflikte und Herausforderungen mittelfristig entstandene „moral distress“, der ebenfalls mit Burnout und Wechselabsichten assoziiert war und während der COVID-19-Pandemie gehäuft auftrat [27].

Als Reaktion wurde im PREPARED-Projekt des Netzwerkes Universitätsmedizin (NUM) der S3-Leitlinien-Prozess „Psychische Gesundheit von Gesundheitspersonal in anhaltenden Krisenlagen“ [14] begonnen. Basierend auf den „WHO Guidelines on Mental Health at Work“ [31] wurde die verfügbare Evidenz neu recherchiert und im Hinblick auf die Resilienzförderung für Krisenlagen in einem von der Arbeitsgemeinschaft Wissenschaftlicher Fachgesellschaften (AWMF) moderierten Konsensusprozess unter Beteiligung 19 wissenschaftlicher Fachgesellschaften sowie von Expertinnen und Experten der Bundesanstalt für Arbeitsschutz und Arbeitsmedizin (BAuA) sowie des Leibniz-Instituts für Resilienzforschung (LIR) Empfehlungen zur Förderung der psychischen Gesundheit am Arbeitsplatz erarbeitet. Neben Forschung zur Implementierung dieser Maßnahmen sollte dabei ein longitudinales, datenbasiertes Monitoring der psychischen Gesundheit von Gesundheitspersonal etabliert werden. In einem multidisziplinären Konzept zur „pandemic preparedness“ wurde deshalb eine stehende Panel-Befragung an Universitätskliniken („MuSe-Sentinels“) vorgeschlagen [22]. So könnten sowohl als Baseline wie auch im Hinblick auf (sich entwickelnde) Krisenlagen Daten zur psychischen Gesundheit des Gesundheitspersonals im Kontext von Infektionsgeschehen, verfügbaren Kapazitäten, Personalqualifikation u. a. gesammelt und diese in agile, zeitnahe Lageeinschätzungen mit einbezogen werden.

## Post-COVID

Bei bisher noch bestehenden Unsicherheiten bezüglich der Prävalenzen leiden möglicherweise bis zu 3,0–13,7 % aller Personen mit einer SARS-CoV-2-Infektion unter nach der Infektion fortbestehenden Long-COVID-Syndromen [29] und 1,2–6,5 % unter einem länger als 12 Wochen anhaltenden Post-COVID-Syndrom [17, 29]. Hier können psychische Symptome wie z. B. Erschöpfung, Belastungsintoleranz, Konzentrationsstörungen und weitere somatische Beschwerden auch Monate nach einer Infektion auftreten oder anhalten und mit relevanten Funktionseinschränkungen verbunden sein [17, 29] und es gibt Hinweise auf Assoziationen zu neu einsetzenden Autoimmunerkrankungen [28]. Derzeit sind zwar an vielen Orten spezialisierte, multiprofessionelle Ambulanzen entstanden, eine S1 Living Guideline existiert [11], es wurde vom Gemeinsamen Bundesausschuss eine Richtlinie zur berufsgruppenübergreifende, koordinierte und strukturierte Versorgung für Versicherte mit Verdacht auf Long-COVID beschlossen [10] und verschiedene Forschungsinitiativen arbeiten an einer breiteren Wissensbasis [5].

Ob diese neu aufgebauten Versorgungsstrukturen jedoch den Bedarfen entsprechen, ist aktuell unklar, es gibt aber Hinweise auf ungedeckte Bedarfe [26]. Da psychische Symptome zentraler Teil der Erkrankung sein können und psychische Erkrankungen daher auch wichtige Differenzialdiagnosen sind, ist dabei eine psychiatrische-psychotherapeutische Beteiligung an multiprofessionellen Angeboten zentral für eine adäquate Versorgung.

## Herausforderungen der psychiatrisch-psychotherapeutischen Versorgung

Die Arbeit von Erdekian et al. zeigt, dass es im ersten Jahr der COVID-19-Pandemie im Bereich der stationären psychiatrisch-psychotherapeutischen Angebote während der beiden Lockdownphasen einen signifikanten Rückgang der Anzahl an Aufnahmen um 20–25 % gab. Auch zwischen den Lockdownphasen gab es keine vollständige Erholung der klinischen Inanspruchnahmeraten [7]. Die wenigen Studien, die auch tagesklinische Angebote untersuchten, zeigen hier einen noch stärkeren Rückgang um 45–70 % [3, 32]. Bezüglich dieser signifikanten Reduktionen der Inanspruchnahme ist wichtig, dass es keine Hinweise auf einen Rückgang von Behandlungsbedarfen gab, sondern für einzelne Indikatoren wie z. B. Suchtmittelkonsum [12] und im längeren Zeitverlauf depressive Symptome in der Allgemeinbevölkerung [30] sogar Zunahmen relevanter Belastungen berichtet wurden. Auch die mittel- und langfristigen Folgen der inadäquaten Behandlung sind noch nicht systematisch abschätzbar, Surveys von Klinikleitungen [32] und ambulant tätigen Psychiaterinnen und Psychiatern [9] wie auch Berichte zu einzelnen Indikationen wie Rückfällen nach unterbrochener Erhaltungs-Elektrokonvulsionstherapie (EKT; [16]) weisen jedoch auf erhöhte Risiken vermeidbarer Exazerbationen, Rückfälle und Chronifizierung hin.

Mehrere Arbeiten deuten darauf hin, dass ausschlaggebend für die Reduktionen der Inanspruchnahme ein Rückgang elektiver Behandlungen war, während Notfallbehandlungen weniger stark zurückgingen [8, 32]. Relevante Gründe von Seiten der Kliniken und psychiatrischen Abteilungen waren Reduktion aufgrund der Anforderungen des Infektionsschutzes, die Veränderungen der Anreizstrukturen durch „Freihalteprämien“ sowie die Notwendigkeit, Isolationskapazitäten für Menschen mit schweren psychischen Erkrankungen und SARS-CoV-2-Infektionen vorzuhalten [32]. Möglicherweise gab es auch seitens der Patientinnen und Patienten eine verstärkte Zurückhaltung insbesondere aus Ängsten vor Infektionen in Gesundheitseinrichtungen [9, 32], wozu jedoch keine Befragungsstudien vorliegen. Rückschlüsse zur Motivation, Behandlungsangebote nicht aufzusuchen, können nur aus diagnosespezifischen Rückgängen der Inanspruchnahme gezogen werden. Im stationären Bereich waren nicht alle Diagnosegruppen in gleichem Ausmaß betroffen, sondern insbesondere Menschen mit affektiven Störungen (u. a. depressive Erkrankungen), Angsterkrankungen und Persönlichkeitsstörungen sowie Suchterkrankungen [7].

Im ambulanten Sektor gab es weniger ausgeprägte Veränderungen der Inanspruchnahme, jedoch Hinweise auf einen erschwerten Zugang, welcher sich in geringeren Zahlen an Neudiagnosen äußerte [7]. In Surveys von Klinikleitungen und ambulant tätigen Psychiaterinnen und Psychiatern wurde zudem deutlich, dass die Angebote des komplementären Versorgungssystem für Menschen mit psychischen Erkrankungen (Tagesstätten, Werkstätten, Wohneinrichtungen wie auch der Rehabilitationssektor) sowie die Zusammenarbeit zwischen den Sektoren erheblich eingeschränkt waren [9, 32]. Ein weiterer Bereich mit erheblichen Einschränkungen waren Visiten von Fachärztinnen und Fachärzten für psychische Erkrankungen in Pflegeheimen und Wohneinrichtungen, vor allem aus Infektionsschutzgründen [9]. Zur Arbeit der öffentlichen Gesundheitsdienste in der Pandemie, also im Psych-Bereich der sozialpsychiatrischen Dienste, können aufgrund fehlender Studien, jedoch auch aufgrund der großen regionalen Heterogenität der Ausgestaltung und Arbeitsweise der Dienste keine Aussagen getroffen werden.

Der Großteil der bislang veröffentlichten Studien deckt lediglich den Zeitraum des ersten Pandemiejahres bis zur zweiten Lockdownphase ab [7]. Daher kann sowohl zu Nachholeffekten wie auch zu den Zunahmen der Inanspruchnahme im Rahmen der verstärkten psychischen Belastung der Allgemeinbevölkerung im weiteren Pandemieverlauf keine Aussage getroffen werden. Es ist zu bedenken, dass Nachholeffekte oder die Zunahme an Bedarfen in Studien, die auf Analysen von Krankenkassenroutinedaten beruhen, auch kaum abzubilden sind, da größere Teile der Kapazitäten des Versorgungssystems gedeckelt sind und aufgrund der Anreizstrukturen außerhalb von Krisenlagen immer mit hoher Auslastung operieren.

## Aufbau einer zeitsensiblen Versorgungssurveillance

Während auf der Ebene der Infektionssurveillance im Laufe der Pandemie Fortschritte gemacht wurden, fehlen bis heute weitgehend zeitnah verfügbare Daten zur Versorgungssurveillance. Signifikante Veränderungen in Versorgungsstrukturen und in der Inanspruchnahme konnten erst mit erheblicher zeitlicher Verzögerung von mehreren Jahren untersucht werden. Zudem fehlen systematische, repräsentative Untersuchungen zu den Folgen dieser Einschränkungen insbesondere auch aus Perspektive betroffener Patientinnen und Patienten, sodass die Auswirkungen der offensichtlich signifikanten Einschränkungen in der Versorgung auf die Gesundheit der Versicherten und mittelbar auch auf die Patientensicherheit unklar bleiben.

Weiterhin bedeutet dies, dass Daten für Lageeinschätzungen und zeitnahe Feedbackschleifen für die Psych-Fächer nicht zur Verfügung standen und weiterhin fehlen. Die Pandemie war dabei keine Ausnahme: Die gleichen Defizite bei der Versorgungssurveillance zeigten sich in Deutschland erneut bei lokalen, klimawandelassoziierten Katastrophen wie der Flutkatastrophe im Ahrtal. Diese fehlende Versorgungssurveillance ist dabei nicht nur ein Problem für die Gesundheitspolitik und die Wissenschaft, sondern auch ein ethisches Problem. Die dafür erforderlichen Daten liegen eigentlich vor, nur ihre zeitsensible Nutzung ist in Deutschland aufgrund organisatorischer und insbesondere datenschutzrechtlicher Hürden blockiert [23]. Diese Nichtnutzung führt dazu, dass Versorgungsdefizite, die konkrete Auswirkungen auf die Gesundheit der Versicherten bis hin zu erhöhter Mortalität haben können [24, 33], nicht oder erst spät erkannt werden.

Wie in [23] detailliert beschrieben, könnte eine integrierte Forschungsdatenplattform ermöglichen, die versorgungsnahen Daten des Forschungsdatenzentrums Gesundheit mit den in den Datenintegrationszentren (DIZ) der Universitätskliniken gesammelten administrativen und medizinischen Klinik- sowie Registerdaten zusammenzuführen. Für die Zukunft könnten hier für die Psych-Fächer dem gerade in Entwicklung befindlichen Kerndatensatzmodul psychische Gesundheit der Medizininformatikinitiative (MII) und dem „minimum dataset“ des Deutschen Zentrums für psychische Gesundheit (DZPG) eine zentrale Rolle zukommen. Auch die ab 2025 für alle Versicherten bereitgestellte elektronische Patientenakte (EPA) mit Opt-Out-Regelung könnte zur Datenverfügbarkeit beitragen, sobald hier strukturierte, standardisierte Daten vorliegen. Eine Herausforderung für die „pandemic and crisis preparedness“ sind dabei Latenzen bei der Datenlieferung und Zusammenführung. In einem Konzept für ein resilientes und agil reagierendes Gesundheitsforschungsnetzwerk zur „pandemic and crisis preparedness“ wurde daher die Etablierung von Universitätskliniken als „MuSe-Sentinel“-Datenlieferanten vorgeschlagen, wo die Zusammenführung relevanter, bereits etablierter und weiterer für das Krisenmanagement erforderlicher Datensätze mit nur geringer Zeitlatenz realisiert werden sollte [22]. Auf die Psych-Fächer entfallen in Deutschland ca. 15 % der Krankenhausbetten und über 20 % der Berechnungs- und Belegungstage [2]. Aufgrund dieser Relevanz für die Patientenversorgung sollten in einem umfassenden Versorgungsmonitoring Indikatoren zur Gesundheitsversorgung von Menschen mit psychischen Erkrankungen mitberücksichtigt werden.

## Sektorübergreifende integrierte Versorgung ermöglichen

Um die Versorgung auch angesichts der verringerten (teil-)stationären Kapazitäten aufrechtzuerhalten und therapeutische Beziehungen nicht abbrechen zu lassen, reagierten sowohl ambulant tätige Psychiaterinnen und Psychiater wie auch viele Kliniken und Abteilungen mit telemedizinischen Angeboten wie Video-Sprechstunden und Video-Therapien. Befragungsstudien von Ärztinnen und Ärzten [9, 32] wiesen jedoch darauf hin, dass die starre Sektorisierung und für Kliniken fehlende Möglichkeiten zur gegenfinanzierten Ambulantisierung von Behandlungen ein zentrales Hindernis darstellten, die von Reduktionen des stationären Versorgungsangebotes betroffenen Gruppen adäquat leitlinienorientiert auch unter den Einschränkungen der Pandemie zu versorgen.

Eine rasche bundespolitische Umsetzung der u. a. von der Regierungskommission zur Krankenhausreform [18] angeregten Maßnahmen zur flexiblen Ambulantisierung psychiatrisch-psychotherapeutischer Behandlungen könnte hier rasch Abhilfe schaffen. Zu diesen Maßnahmen zählen die flächendeckende Einführung des PIA(Psychiatrische Institutsambulanzen)-Einzelleistungs-Abrechnungsmodells sowie die Möglichkeit, ein Regionalbudget (bzw. Globalbudget) nach § 64b Sozialgesetzbuch (SGB) mit Kontrahierungszwang zu etablieren. Kombiniert mit transparenten, datenbasierten Qualitätssicherungsmaßnahmen könnten diese Maßnahmen es Institutionen des stationären Sektors ermöglichen, Behandlungen in Krisenlagen flexibel zu ambulantisieren, ohne zu Behandlungsabbrüchen und in der Folge Verschlechterungen, Exazerbationen und Rückfällen zu führen [32]. Das Beispiel der Pandemie zeigt mittelbar auch über die psychiatrisch-psychotherapeutische Versorgung hinaus, dass Reduktionen stationärer Kapazitäten mit dem Aufbau adäquater ambulanter Alternativen einhergehen müssen und in allen Sektoren flexible Reservekapazitäten (z. B. stationär ausreichend Einzelzimmer für Hygiene-Isolations-Maßnahmen, Möglichkeiten zur Videotherapie etc.) vorgehalten werden sollten.

## Zusammenfassung und Schlussfolgerungen

Zusammenfassend bedeutete die COVID-19-Pandemie in ihrem Verlauf Belastungen für die Allgemeinbevölkerung, besonders jedoch für spezifische Populationen wie Kinder- und Jugendliche, Frauen, sozioökonomische benachteiligte Menschen, Bewohnerinnen und Bewohner von Heim- und Wohneinrichtungen, Gesundheitspersonal und Menschen mit Long‑/Post-COVID-Syndromen. Die psychiatrisch-psychotherapeutische Versorgung war im ersten Jahr der COVID-19-Pandemie insbesondere im stationären Sektor signifikant eingeschränkt, wobei die starre Sektorisierung des deutschen Gesundheitssystems hier vielfach gegenfinanzierte, flexible Alternativangebote verhinderte.

Die Erfahrungen der Pandemie sollte hier von der Politik als weiterer Anlass genommen werden, Strukturen für flexible, integrierte sektorübergreifende Behandlungssequenzen deutschlandweit zu ermöglichen. Für eine verbesserte „pandemic and crisis preparedness“ des Gesundheitsforschungssystems und ein resilienteres Gesundheitssystem sind außerdem einerseits Strukturen wie die Mental Health Surveillance an Institutionen wie dem RKI sehr wichtig. Diese sollte unbedingt erhalten, auskömmlich finanziert [6] und bezüglich der psychischen Gesundheit wichtiger vulnerabler Gruppen wie von Kindern- und Jugendlichen ausgebaut werden. Die Artikel dieses Schwerpunktes zeigen andererseits die Relevanz von Strukturen, welche auch die Gesundheitsversorgung und die Gesundheit des Gesundheitspersonals in Krisenlagen transparent, umfassend und multidisziplinär, also psychische wie körperliche Gesundheit integrierend, zeitsensitiv überschauen, um agil auf Fehlentwicklungen reagieren zu können. In diese Richtung sollten daher komplementäre multidisziplinäre und -perspektivische Strukturen zur „pandemic and crisis preparedness“ im Netzwerk Universitätsmedizin weiterentwickelt werden [22].

## Fazit für die Praxis

Als Lehren aus der Pandemie für eine verbesserte „pandemic and crisis preparedness“ bezüglich psychischer Gesundheit und ein krisenresilienteres psychiatrisch-psychotherapeutisches Versorgungssystem lassen sich zusammenfassen:

- Weiterführung und Ausbau der Nationalen Mental Health Surveillance,
- Strukturen zum Monitoring des psychischen Wohlergehens vulnerabler Gruppen, wie junger Menschen oder Bewohnerinnen und Bewohner von Heim- und Wohneinrichtungen, aber auch besonders exponierter Gruppen wie Gesundheitspersonal,
- Forschung zur Implementierung von Maßnahmen zum Schutz und Erhalt der psychischen Gesundheit von Gesundheitspersonal,
- Strukturen zur Versorgungssurveillance ohne lange Zeitlatenzen und einfachere Datenzugänge sowie Möglichkeiten zur Datenzusammenführung für die Wissenschaft,
- Strukturen zu multiperspektivischen, evidenzbasierten Empfehlungen für wissenschaftliche Diskussion und Politikberatung,
- für die psychiatrisch-psychotherapeutische Versorgung geeignete Finanzierungsmodelle, welche eine flexible, gegenfinanzierte Ambulantisierung der Versorgung (nicht nur) in Krisenlagen ermöglichen, wie z. B. das sächsisch-bayerische PIA-Abrechnungsmodell oder §‑64-Modelle zum Regional- bzw. flexiblen Klinikbudget mit Kontrahierungszwang und verbindlicher transparenter Qualitätssicherung,
- deutschlandweit flächendeckend in Kooperation mit Primärversorgern gestuft operierende, spezialisierte Ambulanzen für Menschen mit Post-COVID-Syndromen.
